# Vehicle State Joint Estimation Based on Lateral Stiffness

**DOI:** 10.3390/s23218960

**Published:** 2023-11-03

**Authors:** Lingxiao Quan, Ronglei Chang, Changhong Guo, Bin Li

**Affiliations:** 1 School of Mechanical Engineering, Yanshan University, Qinhuangdao 066004, China; lingxiao@ysu.edu.cn (L.Q.); changronglei@163.com (R.C.); libin@ysu.edu.cn (B.L.); 2 Hebei Provincial Key Laboratory of Heavy Machinery Fluid Power Transmission and Control, Yanshan University, Qinhuangdao 066004, China

**Keywords:** joint estimator, lateral stiffness, running states of electric vehicles, rear-drive, in-wheel motors, LS-GHCKF/SRGHCKF, double lane change, slalom, robustness

## Abstract

In this study, a vehicle state joint estimation method based on lateral stiffness was applied to estimate the running states of electric vehicles driven by rear-drive, in-wheel motors. Different from the estimation methods used in other research, the joint estimator designed in this study uses the least-squares (LS) algorithm to estimate the lateral stiffness of the front and rear axles of the vehicle, deploying the high-degree cubature Kalman filter algorithm to estimate the vehicle state. We establish a three-degree-of-freedom nonlinear vehicle model with longitudinal velocity, lateral velocity, and yaw rate, and the lateral stiffness of the front and rear axles as the principal parameters. For the low-speed running state of the vehicle, a linearized magic tire model with high fitting accuracy was used to calculate the lateral force of the entire vehicle. The LS algorithm with a forgetting factor was used to design a lateral stiffness estimator to assess the front-axle and rear-axle lateral stiffness of the entire vehicle. The generalized high-degree cubature Kalman filter (GHCKF) algorithm was used to design the vehicle state estimator and further improve the GHCKF algorithm. A vehicle state estimator, using the square root generalized high-degree cubature Kalman filter (SRGHCKF), was designed. Therefore, the joint estimator, comprising a lateral stiffness estimator and a vehicle state estimator, adopts the LS-GHCKF/SRGHCKF algorithm and enables the estimation of the lateral stiffness, the longitudinal velocity, the lateral velocity, and the yaw rate of the entire vehicle during the driving process. A double lane change and slalom simulation were performed to analyze the feasibility and accuracy of the joint estimation algorithm and verify the results of the LS-GHCKF algorithm and the LS-SRGHCKF algorithm. Further, a low-speed driving experiment was carried out for electric vehicles driven by rear in-wheel motors. The inertial navigation system (INS), the global positioning system (GPS), the real-time kinematic (RTK), and an angle sensor were used to collect real-time vehicle data. The results were compared to verify the feasibility of the joint estimator and the progressiveness of the algorithm. The experimental verification and simulation both show that the vehicle state joint estimator, designed based on the LS-GHCKF/SRGHCKF algorithm, can accurately estimate the real-time state of the vehicle. Additionally, the LS-SRGHCKF algorithm shows better effectiveness and robustness than the LS-GHCKF algorithm.

## 1. Introduction

The in-wheel electric vehicle is an emerging automotive technology that integrates the motor and wheel hub to improve the power performance and efficiency of vehicles [1]. The handling stability of in-wheel electric vehicles depends on the torque control of the motor, which requires decision-making based on the state variables of in-wheel electric vehicles. Therefore, the vehicle state variables are important prerequisites for vehicle handling and stability. Additionally, they have a significant impact on the active safety performance of the vehicle [2]. The vehicle status mainly includes the longitudinal velocity, the lateral velocity, the yaw rate, etc. Sensors can be used in the measurement to accurately obtain the above parameters. If all state variables are measured using sensors, the cost is relatively high. Thus, the method of integrating partial low-cost sensor measurement and state estimation is generally used to obtain state variables [3,4]. At present, the neural network method [5], sliding mode observation method [6,7,8], fuzzy logic estimation method [9], Kalman filter method [10,11], etc., are widely used in vehicle state estimation algorithms. The neural network method requires a large number of samples and possesses strict requirements. The sliding mode observation method requires state estimation to strictly follow the motion of the sliding mode surface. The weighting coefficients of fuzzy logic estimation are difficult to determine. The Kalman filter algorithm has the advantage of recursive iteration, which can effectively suppress noise and improve system accuracy. Therefore, the Kalman filter method is widely used. On the basis of classical Kalman filter algorithms, an extended Kalman filter (EKF), unscented Kalman filter (UKF), and cubature Kalman filter (CKF) were developed. These algorithms are widely used in the field of vehicle state estimation.

References [12,13] integrated the EKF algorithm with other algorithms for state estimation. Reference [12] combined EKF with an improved radial basis function (RBF) neural network to jointly estimate the centroid sideslip angle and road adhesion coefficient. Compared with the results obtained when using the EKF algorithm or RBF algorithm alone, the joint estimation algorithm had higher accuracy [12]. Reference [13] combined EKF with a limited-memory filter to develop a limited-memory adaptive extended Kalman filter (LM-AEKF). Like reference [12], the fused algorithm has significant advantages in terms of filtering stability and estimation accuracy [13]. A dual unscented Kalman filter (DUKF) algorithm was proposed in references [14,15]. Reference [14] analyzed and derived the local observability of the DUKF observer through the theory of differential geometry, comparing the estimation results with those of the dual extended Kalman filter (DEKF) [14]. The DUKF algorithms in reference [15] jointly observed the vehicle state: one of the two filters observed the vehicle speed, the sideslip angle of the body’s center of mass, and other states, while the other one observed the vehicle inertia parameters [15]. The DUKF proposed in references [14,15] received solid observation results. References [16,17] proposed an improved algorithm based on the CKF algorithm. Reference [16] adopted a fuzzy adaptive robust cubature Kalman filter (FARCKF) to estimate the sideslip angle and tire stiffness. At the same time, the least-square method was used to update the model parameters of FARCKF [16]. Reference [17] proposed the adaptive square-root cubature Kalman filter (ASCKF) algorithm that adaptively adjusts the model parameters of ASCKF [17]. Through simulations and experiments, the improved cubature Kalman algorithm proposed in references [16,17] was found to be feasible and to have reliable results. Reference [18] proposed a vehicle state estimation method that superimposes Kalman filter algorithms and accurately estimates the vehicle state parameters [18]. Reference [19] used an EKF algorithm to estimate the state of the vehicle. According to the random weighting theory, the weighting coefficient subject to Dirichlet distribution is designed to further improve the accuracy of the estimation [19]. In reference [20], in order to effectively estimate the vehicle state with the non-Gaussian noise, the maximum correntropy criterion was combined with an adaptive extended Kalman filter (AEKF) [20]. Reference [21] proposed an adaptive filtering algorithm that combines UKF and a genetic algorithm to achieve adaptive process noise and measurement noise and to accurately estimate the operating status of the vehicle [21]. To improve the accuracy of state parameter estimation for distributed drive electric vehicles, a UKF algorithm combined with the Huber method was proposed in reference [22]. This algorithm improves the robustness of the observer, reflects vehicle status in real-time, effectively suppresses the influence of errors and noise, and achieves high observation accuracy [22]. A traditional Kalman filter algorithm has poor accuracy and robustness in solving the problem of non-Gaussian noise. Reference [23] presented a robust hierarchical estimation scheme for the vehicle driving state based on the maximum correntropy square-root cubature Kalman filter (MCSCKF) and achieved good results [23]. The adaptive volume particle filter (ACPF) proposed in reference [24] estimates key state variables such as vehicle roll angle and center-of-mass roll angle [24]. The least-squares method can effectively and concisely solve nonlinear problems and improve the rate of convergence. As such, it is often used in the field of parameter detection and variable estimation [25]. In reference [26], the LS method and fuzzy adaptive extended Kalman algorithm are used to estimate the vehicle state and achieve good results [26]. The above literature involves EKF, UKF, and CKF. Through literature analysis, the comparison of the three Kalman filtering algorithms is as follows (Table 1):

Therefore, after the above comparative analysis, and factoring in computational efficiency and accuracy, it can be determined that CKF is more suitable for complex nonlinear systems. GHCKF is derived from CKF. The GHCKF algorithm can deal with more complex systems than CKF, including nonlinear and non-Gaussian systems [27,28,29,30]. In practical applications, as the vehicle system is a complex nonlinear system, the use of a high-order cubature Kalman filtering algorithm can better process nonlinear information and improve filtering accuracy. This article uses the LS algorithm to estimate the lateral stiffness of vehicles in real-time, rather than traditional methods such as testing and table lookup. Based on lateral stiffness estimation, the vehicle state is estimated using nonlinear three-degrees-of-freedom. On this basis, the GHCKF algorithm is used to estimate the vehicle state. Further, we combine the square root method with GHCKF to develop a new SRGHCKF vehicle state estimator. In theory, the SRGHCKF algorithm has better robustness and effectiveness than the GHCKF algorithm. Under the framework of the LS-GHCKF/SRGHCKF joint estimation method, the dynamic behavior of vehicles is modeled and predicted while filtering out the influence of sensor noise and uncertain factors to improve vehicle handling performance and safety. This article uses experimental and simulation methods to verify whether the joint estimation method of LS-GHCKF/SRGHCKF can better handle complex nonlinear systems such as vehicles. If this method can provide an optimal estimation of the vehicle state, it indicates that the LS-GHCKF/SRGHCKF joint estimation method has important application value. At the same time, it is also necessary to prove that the LS-SRGHCKF algorithm is a more efficient and reliable vehicle state estimation algorithm compared to the LS-GHCKF algorithm.

## 2. Design of Vehicle State Joint Estimator

**Figure 1:**
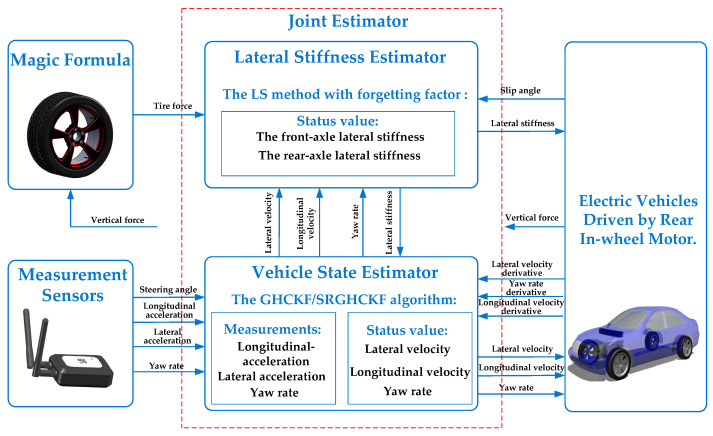
The schematic diagram of joint estimation.

The vehicle state estimation topic studied in this paper consists of five parts: the model of an electric vehicle driven by a rear in-wheel motor; measurement sensors; a magic formula; a lateral stiffness estimator, and a vehicle state estimator. The lateral stiffness estimator and the vehicle state estimator form a joint estimator as shown in Figure 1.

The principle of the joint estimation of vehicle states is as follows: the variables in the lateral stiffness estimator and vehicle state estimator modules are state variables, and the transmission variables indicated by the arrows are system parameters. When the state variables leave the estimator as transmission variables, they are also considered to be parameters. The sensors measure parameters such as steering wheel angle, longitudinal acceleration, lateral acceleration, and yaw rate. These are input into the vehicle state estimator for calculation. The magic formula receives the vertical force parameters of the vehicle model and calculates the lateral force of the vehicle. Then, they are input into a lateral stiffness estimator. The vehicle model receives the estimated values of the joint estimator, calculates the sideslip angles, longitudinal velocity derivative, lateral velocity derivative, and yaw velocity derivative, and then returns to the joint estimator for calculation. The lateral stiffness estimator and vehicle state estimator exchange parameters such as lateral stiffness, longitudinal velocity, lateral velocity, and yaw rate. Then, the lateral stiffness estimator and vehicle state estimator use advanced algorithms to calculate these input variables, respectively, to achieve the estimated results of the electric vehicle driven by the rear-drive, in-wheel motor.

### 2.1. The Vehicle State Estimator

A vehicle state estimator was designed based on a three-degree-of-freedom vehicle model using the GHCKF algorithm. Then, a more advanced SRGHCKF algorithm was developed for estimating the vehicle state.

#### 2.1.1. The Linear Two-Degree-of-Freedom Vehicle Model

**Figure 2:**
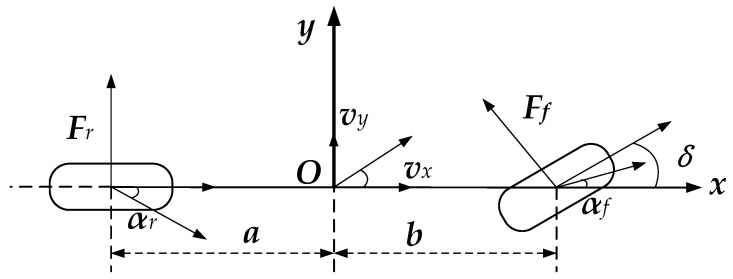
The linear two-degree-of-freedom vehicle model.

A linear two-degree-of-freedom model for the entire vehicle was established, and the following assumptions were made: (1) ignoring the suspension effect, it was assumed that the vehicle only moves parallel to the ground; (2) the origin of the vehicle coordinate system of the entire vehicle model coincides with the center of mass of the vehicle; (3) the influence of longitudinal rolling resistance on vehicle status is ignored. The linear two-degree-of-freedom model is shown in Figure 2.

The absolute acceleration of the vehicle’s center of mass on the *y* axis of the vehicle coordinate system:

(1) $$a_{y}=\frac{(aC_{f}-bC_{r})\omega_{r}}{mv_{x}}+\frac{(C_{f}+C_{r})\beta }{m}-\frac{C_{f}\delta }{m}$$

In the above formula, *a_y_* is the lateral acceleration of the vehicle; *β* is the sideslip angle of the center of mass; *ω_r_* is the yaw rate; *C_f_* is the vehicle’s front-axle lateral stiffness; *C_r_* is the vehicle’s rear-axle lateral stiffness; *m* is the mass of the entire vehicle; *a* is the distance from the center of mass to the front axle; *b* is the distance from the center of mass to the rear axle; and Δ is the front wheel steering angle.

Moment balance equation around the *z* axis:

(2) $${\dot{\omega }}_{r}=\frac{(aC_{f}-bC_{r})\beta }{I_{z}}+\frac{(a^{2}C_{f}+b^{2}C_{r})\omega_{r}}{I_{z}v_{x}}-\frac{aC_{f}\delta }{I_{z}}$$

In the above formula, *I_z_* is the moment of inertia of the entire vehicle around the *z* axis; *v_x_* is the longitudinal velocity of the center of mass.

The vehicle centroid sideslip angle:

(3) $$\beta =\arctan(\frac{v_{y}}{v_{x}})\approx \frac{v_{y}}{v_{x}}$$

In the above formula, *v_y_* is the lateral velocity of the center of mass.

#### 2.1.2. The Nonlinear Three-Degree-of-Freedom Vehicle Model

**Figure 3:**
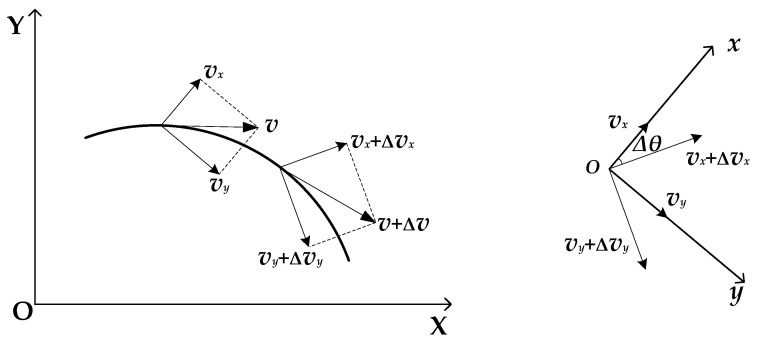
Analysis of vehicle motion in vehicle coordinate system.

Because vehicles do not travel in a fixed straight line during the actual driving process, various operating conditions such as turning or overtaking can cause the vehicle to experience bumps or roll. Therefore, when a vehicle model is established, the yaw and sideslip situations are considered. Additionally, the vehicle also has nonlinear characteristics when traveling longitudinally. Therefore, based on the linear two-degree-of-freedom vehicle model, a nonlinear three-degree-of-freedom vehicle model with specific yaw, lateral, and longitudinal characteristics was established [31]. We derived a longitudinal kinematic formula based on Figure 3 [32].

Velocity variation along the *x* axis direction:

(4) $$\begin{array}{l} (v_{x}+\Delta v_{x})\cos\Delta \theta -(v_{y}+\Delta v_{y})\sin\Delta \theta -v_{x}\\ =v_{x}\cos\Delta \theta +\Delta v_{x}\cos\Delta \theta -v_{y}\sin\Delta \theta -\Delta v_{y}\sin\Delta \theta -v_{x} \end{array}$$

In the above formula, Δ*v_x_* is the longitudinal velocity increment of the *x* axis in the vehicle coordinate system; Δ*v_y_* is the lateral velocity increment of the *y* axis in the vehicle coordinate system; and Δ*θ* is the rotation angle increment of the vehicle coordinate system.

Considering a very small Δ*θ* and ignoring second-order trace amounts:

(5) cosΔθ≈1,sinΔθ≈Δθ

(6) $$\begin{array}{l} v_{x}\cos\Delta \theta +\Delta v_{x}\cos\Delta \theta -v_{y}\sin\Delta \theta -\Delta v_{y}\sin\Delta \theta -v_{x}\\ =v_{x}+\Delta v_{x}-v_{y}\cdot \Delta \theta -\Delta v_{y}\cdot \Delta \theta -v_{x}\\ =\Delta v_{x}-v_{y}\cdot \Delta \theta \end{array}$$

Divide by Δ*t* and take the limit. The longitudinal acceleration of the vehicle’s center of mass on the *x* axis of the vehicle coordinate system is as follows:

(7) $$a_{x}=\frac{\Delta v_{x}-v_{y}\cdot \Delta \theta }{\Delta t}=\frac{dv_{x}}{dt}-v_{y}\frac{d\theta }{dt}={\dot{v}}_{x}-v_{y}\cdot \omega_{r}$$

In the above formulae, *a_x_* is the longitudinal acceleration of the vehicle.

Similarly, the lateral acceleration of the vehicle’s center of mass on the *x* axis of the vehicle coordinate system can be obtained:

(8) $$a_{y}={\dot{v}}_{y}+v_{x}\cdot \omega_{r}$$

#### 2.1.3. The Vehicle State Estimation Model

According to the structure of the vehicle state joint estimator designed in this article, the vehicle state is estimated based on the nonlinear vehicle dynamics model. For general nonlinear systems, the state estimation model can be expressed as:

(9) $$\left\{\begin{array}{l} \dot{x}(t)=f(x(t),u(t),w(t))\\ \dot{z}(t)=h(x(t),u(t),v(t)) \end{array}\right.$$

In the above equations, **w**(*t*) and **v**(*t*), respectively, represent the process noise and the measurement noise matrices of the system, which follow a Gaussian distribution and are independent of each other.

The state vector of the vehicle state estimator is defined as **x***_c_*(*t*) = [*v_x_,v_y_,ω_r_*]^T^. The measurement vector of the vehicle state estimator is defined as **z***_c_*(*t*) = [*a_y_,ω_r_*]^T^. From the parameter flow direction shown in Figure 1, it can be seen that the input vector of the vehicle state estimator not only includes the measurement parameters of the steering angle, longitudinal acceleration, lateral acceleration, and yaw rate of the sensors but also includes the estimated lateral stiffness of the front and rear axles by the lateral stiffness estimator. As such, the input vector of the vehicle state estimator is defined as **u***_c_*(*t*) = [Δ*,a_x_,a_y_,ω_r_,C_f_,C_r_*]^T^.

By combining nonlinear vehicle dynamics models (1), (2), (3), (7), and (8), the state equation of the vehicle estimator is established:

(10) $$\left[\begin{array}{l} {\dot{\omega }}_{r}\\ {\dot{v}}_{y} \end{array}\right]=\left[\begin{array}{cc} \frac{\left(aC_{f}+bC_{r}\right)}{I_{z}v_{x}} & \frac{\left(aC_{f}+bC_{r}\right)}{I_{z}v_{x}}\\ \frac{\left(aC_{f}-bC_{r}-mv_{x}^{2}\right)}{mv_{x}} & \frac{\left(C_{f}+C_{r}\right)}{mv_{x}} \end{array}\right]\left[\begin{array}{c} \omega_{r}\\ v_{y} \end{array}\right]-\left[\begin{array}{c} \frac{aC_{f}\delta }{I_{z}}\\ \frac{C_{f}\delta }{m} \end{array}\right]$$

(11) $${\dot{v}}_{x}=v_{y}\cdot \omega_{r}+a_{x}$$

Combining the nonlinear vehicle dynamics model (1), the measurement equation of the vehicle estimator is established:

(12) $$\left[\begin{array}{c} a_{y}\\ \omega_{r} \end{array}\right]=\left[\begin{array}{cc} \frac{\left(aC_{f}-bC_{r}\right)}{mv_{x}} & \frac{\left(C_{f}+C_{r}\right)}{mv_{x}}\\ 1 & 0 \end{array}\right]\left[\begin{array}{c} \omega_{r}\\ v_{y} \end{array}\right]-\left[\begin{array}{c} \frac{C_{f}\delta }{m}\\ 0 \end{array}\right]$$

#### 2.1.4. Design Generalized High-Degree Cubature Kalman Estimator

This article adopts the GHCKF algorithm [33]: this is concise in form, computationally efficient, and has better scalability. It considers discrete nonlinear systems such as in Equation (9), which limit the nonlinear filtering integral equation to the real number field *R*^n^:

(13) $$I(g)=\int_{R^{n}}g(x)\exp(-x^{T}x)dx$$

Equation (13) is transformed into the standard Gaussian distribution. Then, the following formula is obtained [34]:

(14) $$I(g)=\frac{1}{\sqrt{\pi^{n}}}\int_{R^{n}}g(\sqrt{2}x)\exp(-x^{T}x)dx=\sum_{i=1}^{2n^{2}+1}\rho_{i}g(\eta_{i})$$

Among them, the cubature points *ξ_i_* and the weight values *w_i_* are:

(15) $$\eta_{i}=\left\{\begin{array}{l} {\left[0\right]}_{i},i=1;\\ {\left[\sqrt{3}\right]}_{i},i=2,\cdots ,2n+1;\\ {\left[\sqrt{3},\sqrt{3}\right]}_{i},i=2n+2,\cdots ,2n^{2}+1. \end{array}\right.$$

(16) $$\rho_{i}=\left\{\begin{array}{l} (1-(7-n)n/18),i=1;\\ (4-n)/18,i=2,\cdots ,2n+1;\\ 1/36,i=2n+2,\cdots ,2n^{2}+1. \end{array}\right.$$

In the above equations, *n* is the number of state variables. Equations (14)–(16) are then implemented into the CKF framework to obtain the standard GHCKF algorithm. The GHCKF algorithm for the vehicle state estimation is as follows:

(1) Initialize state-estimated values and error covariance:

(17) $${\overset{⌢}{x}}_{k}=x_{0};\;P_{k}=P_{0}$$

(2) Calculate cubature points *x_k_^i^* (*i* = 1, 2, …, 2*n*^2^ + 1):

(18) $$S_{k}=Cholesky(P_{k})$$

(19) $$x_{k}{}^{i}=S_{k}\eta_{i}+{\hat{x}}_{k}$$

(3) Calculate cubature points propagated using the state equation *x_k|k_*_+1_*^i^*:

(20) $$x_{k+1|k}{}^{i}=f(x_{k}^{i})$$

(4) Calculate the state estimation value at the current moment $\hat{x}$*_k|k_*_+1_*^i^*:

(21) $${\hat{x}}_{k+1|k}=\sum_{1}^{2n^{2}+1}\rho_{i}x_{k+1|k}{}^{i}$$

(5) Calculate the prediction error covariance *P_k|k_*_+1_:

(22) $$P_{k+1|k}=\sum_{1}^{2n^{2}+1}\rho_{i}(x_{k+1|k}{}^{i}-{\hat{x}}_{k+1|k}){\left(x_{k+1|k}{}^{i}-{\hat{x}}_{k+1|k}\right)}^{T}+Q_{k}$$

In this equation, *Q_k_* is the process noise covariance.

(6) Calculate cubature points *x_k_*_+1_*^i^* (*i* = 1, 2, …, 2*n*^2^ + 1):

(23) $$S_{k+1}=Cholesky(P_{k+1|k})$$

(24) $$x_{k+1}{}^{i}=S_{k+1}\eta_{i}+{\hat{x}}_{k+1|k}$$

(7) Calculate cubature points propagated using the measurement equation *z_k|k_*_+1_*^i^*:

(25) $$z_{k+1|k}{}^{i}=h(x_{k+1}^{i})$$

(8) Calculate the measurement prediction value at the current moment $\hat{z}$*_k|k_*_+1_*^i^*:

(26) $${\hat{z}}_{k+1|k}=\sum_{1}^{2n^{2}+1}\rho_{i}z_{k+1|k}{}^{i}$$

(9) Calculate measurement error covariance *P_k_*_+1_*^zz^* and cross-correlation covariance *P_k_*_+1_*^xz^*:

(27) $$P_{k+1}^{zz}=\sum_{1}^{2n^{2}+1}\rho_{i}(z_{k+1|k}{}^{i}-{\hat{z}}_{k+1|k}){\left(z_{k+1|k}{}^{i}-{\hat{z}}_{k+1|k}\right)}^{T}+R_{k}$$

(28) $$P_{k+1}^{xz}=\sum_{1}^{2n^{2}+1}\rho_{i}(x_{k+1|k}{}^{i}-{\hat{x}}_{k+1|k}){\left(z_{k+1|k}{}^{i}-{\hat{z}}_{k+1|k}\right)}^{T}$$

In this equation, *R_k_* is the measurement noise covariance.

(10) Update the filter gain matrix **K***_k_*_+1_, the state variable matrix ${\hat{x}}_{k+1}$, and the error covariance matrix **P***_k_*_+1_:

(29) $$K_{k+1}=P_{k+1}^{xz}{\left(P_{k+1}^{zz}\right)}^{-1}$$

(30) $${\hat{x}}_{k+1}={\hat{x}}_{k+1|k}+K_{k+1}(z_{k}-{\hat{z}}_{k+1|k})$$

(31) $$P_{k+1}=P_{k+1|k}-K_{k+1}P_{k+1}^{zz}K_{k+1}{}^{T}$$

In this formula, **z***_k_* is the measured value matrix at the current time.

#### 2.1.5. Design Square Root Generalized High-Degree Cubature Kalman Estimator

In order to further explore the progressiveness of the joint estimator, the SRGHCKF estimation algorithm is proposed by combining the square root theory with the GHCKF algorithm. In the SRGHCKF algorithm, the main function of the square root is to optimize the calculation process of the Kalman filter and improve the computational efficiency. The SRGHCKF algorithm utilizes the square root to approximate the iterative process of the state estimation and covariance estimation of the Kalman filter, thereby avoiding the complexity of directly solving the Kalman equation.

Specifically, the SRGHCKF algorithm uses iterative equations in the square root form for state estimation and covariance estimation, rather than directly solving the Kalman equation. This iterative method can use the square root of the matrix to approximate the inverse of the matrix, thereby avoiding the computational complexity and numerical stability issues of directly solving the inverse of the matrix. In addition, the SRGHCKF algorithm also adopts a volume update method, which combines the iterative process of state estimation and covariance estimation, thereby improving computational efficiency.

The square root method process is as follows [35,36]:

Calculate the square root factor of the prediction error covariance *S_k_*_+1_:

(32) $$X_{k+1|k}=\frac{1}{\sqrt{2n^{2}+1}}\left[\sum_{1}^{2n^{2}+1}\left(x_{k+1|k}{}^{i}-{\hat{x}}_{k+1|k}\right)\right]$$

(33) $$S_{Q_{k}}=chol(Q_{k})$$

(34) $$S_{k+1}=qr([X_{k+1|k},S_{Q_{k}}])$$

Calculate the covariance of innovation *S_k_*_+1_*^zz^*:

(35) $$Z_{k+1|k}=\frac{1}{\sqrt{2n^{2}+1}}\left[\sum_{1}^{2n^{2}+1}\left(z_{k+1|k}{}^{i}-{\hat{z}}_{k+1|k}\right)\right]$$

(36) $$S_{R_{k}}=chol(R_{k})$$

(37) $$S_{k+1}^{zz}=qr([Z_{k+1|k},S_{R_{k}}])$$

Calculate the cross-correlation covariance *S_k_*_+1_*^xz^*:

(38) $$X_{k+1}=\frac{1}{\sqrt{2n^{2}+1}}\left[\sum_{1}^{2n^{2}+1}\left(x_{k+1}{}^{i}-{\hat{x}}_{k+1|k}\right)\right]$$

(39) $$S_{k+1}^{xz}=X_{k+1}Z_{k+1|k}^{T}$$

Update the filter gain matrix **K***_k_*_+1_, the square root factor of the error covariance matrix **S***_k_*_+1_:

(40) $$K_{k+1}=S_{k+1}^{xz}{\left(S_{k+1}^{zz}S_{k+1}^{zz}{}^{T}\right)}^{-1}$$

(41) $$S_{k+1}=qr([X_{k+1}-K_{k+1}Z_{k+1|k},K_{k+1}S_{R_{k}}])$$

**Figure 4:**
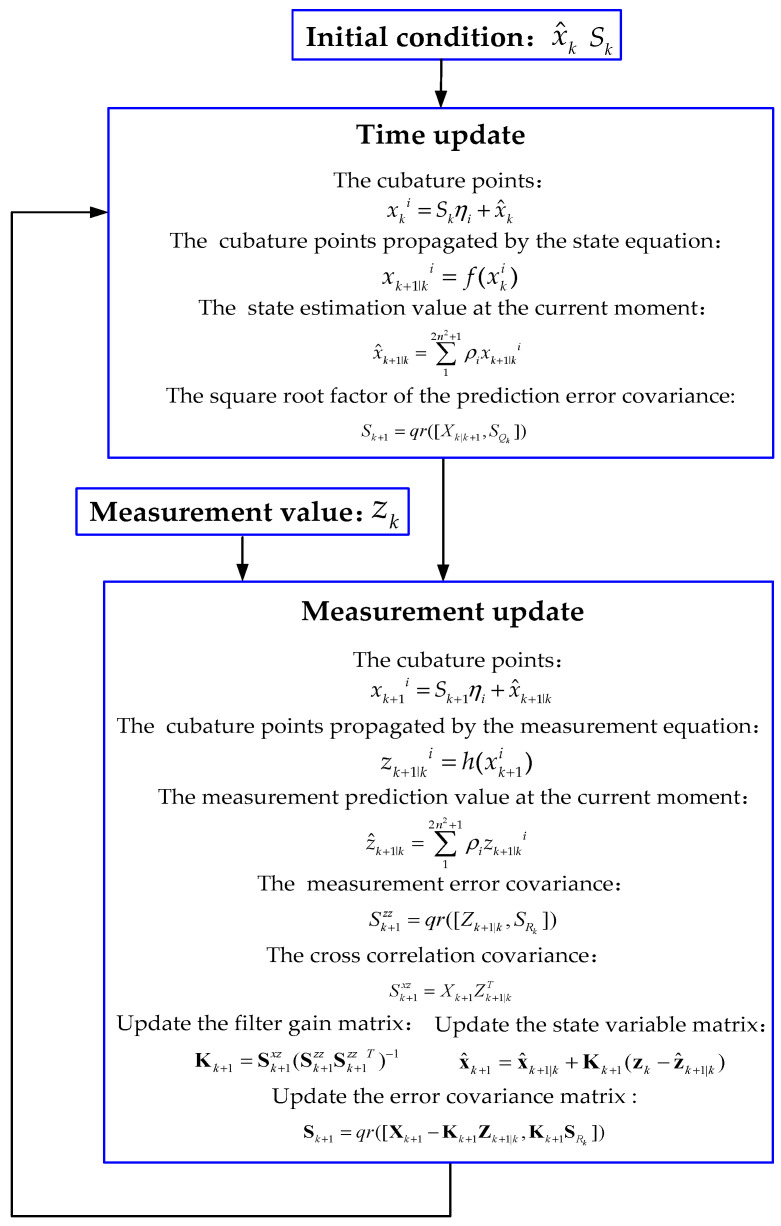
The SRGHCKF algorithm flowchart.

Replace Equations (22) and (23) with Equations (32)–(34), Equations (27) and (28) with Equations (35)–(39), Equation (29) with Equation (40), and Equation (31) with Equation (41), and obtain the SRGHCKF algorithm process, as shown in Figure 4.

### 2.2. The Lateral Stiffness Estimator

The estimation of lateral stiffness is based on the magic formula, using the least-squares method with the forgetting factor to estimate the vehicle’s front- and rear-axle lateral stiffness online. Due to the fact that the estimation of lateral stiffness is within the linear range of the tire, this estimation method is generally applicable to low-speed driving conditions.

#### 2.2.1. Magic Formula

The “magic formula” uses the combination formula of specific trigonometric functions to fit experimental tire data. With a set of formulae in the same form, the longitudinal force, the lateral force, the righting moment, the reversing moment, the rolling resistance, and the working conditions under the combined action of the longitudinal force and lateral force can be completely expressed. The magic formula has strong uniformity, and the parameters that require fitting have clear physical meanings. Moreover, the magic formula is based on test data. It can show high accuracy within the test range and even be used to a certain extent beyond the limit value. The magic formula has high fitting accuracy but requires a large amount of calculation. As such, it is more suitable for use in product design, automobile dynamic simulation, test comparison, and other fields that require an accurate description of tire mechanical properties [37,38].

According to the magic formula, under a single operating condition of pure lateral force, the relationship between the lateral force, the sideslip angle, and the vertical ground load *F_z_* of the tire is:

(42) $$\left\{\begin{array}{l} y(x)=D_{y}\sin\left\{C_{y}\arctan\left[B_{y}x-E_{y}(B_{y}x-\arctan B_{y}x)\right]\right\}\\ Y(X)=y(x)+S_{v}\\ x=\alpha +S_{h} \end{array}\right.$$

where

(43) $$\begin{array}{l} D=a_{1}F_{zi}^{2}+a_{2}F_{zi},\;E=a_{7}F_{zi}^{2}+a_{8}F_{zi}+a_{9},\;S_{h}=a_{10}\cdot \gamma \\ S_{v}=(a_{11}F_{zi}^{2}+a_{12}F_{zi})\cdot \gamma ,\;B\cdot C\cdot D=(a_{3}F_{zi}^{2}+a_{4}F_{z})\cdot e^{-a_{12}\cdot F_{zi}} \end{array}$$

In the formula, *Y*(*x*) is the lateral force, including the front-tire lateral force *F_f_* and the rear-tire lateral force *F_r_*; *α* is the sideslip angle, including the front-axle sideslip angle *α_f_* and the rear-axle sideslip angle *α_r_*; *D_y_* is the peak factor; *B_y_* is the stiffness factor; *C_y_* is the curve shape factor; *E_y_* is the curvature factor of the curve; *S_h_* represents the horizontal drift of the curve; *S_v_* represents the vertical drift of the curve; *a*_1_*…a*_12_ are the fitting parameters.

#### 2.2.2. The Lateral Stiffness Estimation Model

As shown in Figure 1, in order to calculate the tire force, the vertical load on each axis must be calculated first. For the vehicle model shown in Figure 2, ignoring the lateral load transfer of the tires, the vertical load on the front and rear axles is:

(44) $$\begin{array}{l} F_{zf}=\frac{1}{\left(a+b\right)}(mgb-mh_{G}a_{x})\\ F_{zr}=\frac{1}{\left(a+b\right)}(mga+mh_{G}a_{x}) \end{array}$$

In the formula, *F_zi_* is the vertical ground load, and *i* takes *f* and *r*, representing the front tire and rear tire, respectively; and *h_G_* is the vehicle centroid height.

When the tire sideslip angle is small, there is a linear relationship between the tire lateral force and the front- and rear-axle sideslip angle under different loads. The tire lateral force can be approximated as a linear function of the front- and rear-axle sideslip angle:

(45) $$\begin{array}{l} F_{f}=C_{f}\alpha_{f}\\ F_{r}=C_{r}\alpha_{r} \end{array}$$

Due to the fact that the linearized tire model only has high fitting accuracy when the tire sideslip angle is small, and considering the monorail vehicle model shown in Figure 2, the method of small angle assumptions can approximate the front- and rear-axle sideslip angles:

(46) $$\begin{array}{l} \alpha_{f}=\arctan(\frac{v_{y}+a\omega_{r}}{v_{x}})-\delta \approx \frac{v_{y}+a\omega_{r}}{v_{x}}-\delta \\ \alpha_{r}=\arctan(\frac{v_{y}-b\omega_{r}}{v_{x}})\approx \frac{v_{y}-b\omega_{r}}{v_{x}} \end{array}$$

When the lateral acceleration *a_y_* ≤ 0.4 g and the tire sideslip angle *α_y_* ≤ 6°, a magic tire linearization model has high fitting accuracy for conventional tires. At this point, Equation (45) is applied to calculate the front- and rear-axle lateral stiffness within the linear range of the tire.

#### 2.2.3. Design Lateral Stiffness Estimator

Tire linear lateral stiffness is an important parameter for linearizing a vehicle model and reflects the stability characteristics of the vehicle in the linear region of the tire. This parameter often changes with different driving conditions and road conditions. The real-time estimation of tire linear lateral stiffness can improve the adaptability of running vehicles to road surfaces with different adhesion coefficients. This article uses the LS method with a forgetting factor to estimate front- and rear-axle lateral stiffness [39,40].

According to Equation (45), the linear discrete regression equation is established, including the tire lateral force with noise and the linear lateral stiffness:

(47) $$F_{y}(k)=C(k)\alpha (k)+\psi (k)$$

In the above equation, **F***_y_*(*k*) is the lateral force matrix, **C**(*k*) is the lateral stiffness matrix, **α**(*k*) is the sideslip angle matrix, and **ψ**(*k*) is the noise matrix.

The principle of model parameter estimation based on the LS method can be expressed as follows: in the studied historical data window, if the square difference between the actual value and the estimated value is minimized, then the cost function *J* can be expressed as:

(48) $$J=\sum_{i=1}^{N}\lambda^{N-i}\left[F_{y}(i)-\overset{⌢}{C}(i)\alpha (i)\right]$$

Solve the cost function, and obtain the recursive least-squares formula with the forgetting factor *λ*:

(49) $$P(k)={\left[\sum_{i=1}^{N}\lambda^{N-i}\left[\alpha (i)\alpha^{T}(i)\right]\right]}^{-1}$$

(50) $$P(k+1)=\lambda^{-1}\left[P(k)-K(k+1)\alpha^{T}(k+1)P(k)\right]$$

(51) $$K(k+1)=P(k)\alpha (k+1){\left[\lambda +\alpha^{T}(k+1)P(k)\alpha (k+1)\right]}^{-1}$$

(52) $$\overset{⌢}{C}(k+1)={\overset{⌢}{C}}_{y}(k)+K(k+1)\left[F_{y}(k+1)-\alpha^{T}(k+1)\overset{⌢}{C}(k)\right]$$

Among them, **K**(*k* + 1) is the correction vector matrix; **P**(*k* + 1) is the covariance matrix of the error; and *λ* is the forgetting factor, 0 < *λ* < 1. The forgetting factor can ensure that Equation (52) does not degrade its correction ability as the sampled data increase; *N* is the step size of the operation time window, and **C**($\hat{k}$) represents the estimated matrix of the lateral stiffness.

## 3. Simulation Analysis

A joint simulation platform is built based on the nonlinear vehicle dynamics model and the proposed algorithms to jointly simulate the vehicle state joint estimator. As the lateral stiffness estimator proposed in this article is designed for the linear deformation zone of the tire, the vehicle speed in the simulation is set to a low speed, and double lane change and slalom are selected to verify the effectiveness of the joint estimator. A comparison is made between the effectiveness of the LS-GHCKF algorithm and the LS-SRGHCKF algorithm.

### 3.1. Double Lane Change Simulation

**Figure 5:**
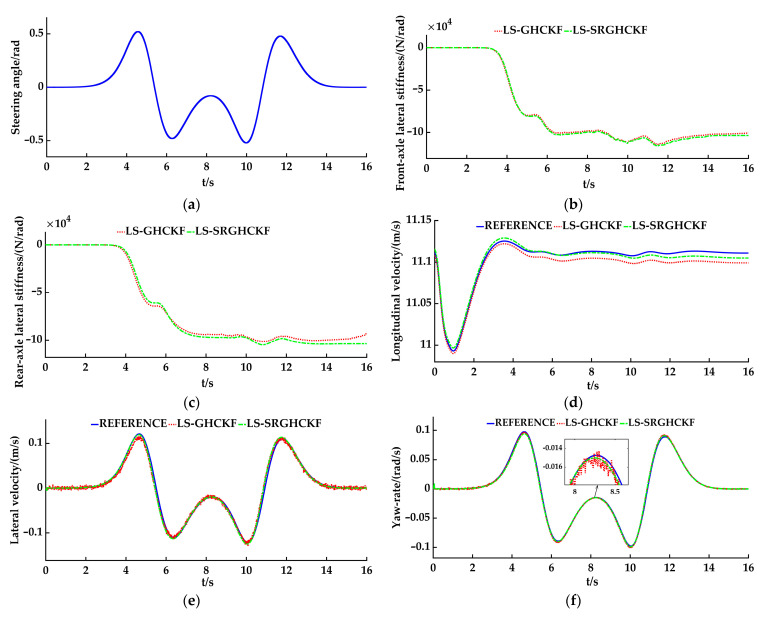
The simulation results of the double lane change: (**a**) the steering angle; (**b**) the front-axle lateral stiffness; (**c**) the rear-axle lateral stiffness; (**d**) the longitudinal velocity; (**e**) the lateral velocity; (**f**) the yaw rate.

The low-speed double lane change simulation working conditions were as follows: The vehicle speed is set to 40 km/h and the road adhesion coefficient *μ* is set to 0.85. The simulation step size is set at 0.005 s. The setting of the initial condition is as follows: the error covariance matrix **P** = 0.1 × eye(3), the process noise covariance matrix **Q** = 0.01 × eye (3), and the measurement noise covariance matrix **R** = 0.01 × eye (2). The simulation results are shown in Figure 5a–f. The blue solid line represents the reference value, the red dashed line represents the estimated value of the LS-GHCKF algorithm, and the green dotted line represents the estimated value of the LS-SRGHCKF algorithm.

Analysis of the simulation results: Figure 5a shows the steering wheel angle-change curve during the vehicle’s running process along the double lane change path. As shown in Figure 1, the lateral stiffness estimator can estimate the front- and rear-axle lateral stiffness after receiving the state variables, such as *v_x_*, *v_y_*, and *ω_r_* from the vehicle state estimator. Figure 5b and Figure 5c, respectively, show the front- and rear-axle lateral stiffness curves based on the state variables of the vehicle state estimators adopting the LS-GHCKF and LS-SRGHCKF algorithms. As shown in the figures, the estimated values of lateral stiffness in the two algorithmic environments are similar in size, and the trends of the data are basically the same. The lateral stiffness changes with the driving time, especially during steering. After stable operation, overall, the average absolute value of the front-axle lateral stiffness of the two algorithms is about 103,000 N/rad, the average absolute value of the rear-axle lateral stiffness is about 95,000 N/rad, and the value of the rear-axle lateral stiffness is smaller than the value of the front-axle lateral stiffness. For the entire vehicle model used for simulation, the ratio of the distance from the center of mass to the rear axle to the distance from the center of mass to the front axle is 1.87. Thus, it can be calculated that the front-axle vertical load is greater than the rear-axle vertical load. According to automotive theory, within a certain range, the lateral stiffness increases with an increase in vertical load; therefore, the front-axle lateral stiffness is greater than the rear-axle lateral stiffness. When the vehicle turns, the vertical loads on the front and rear axles undergo a sudden change, and the lateral stiffness also changes accordingly. Therefore, the above simulation results are in line with theoretical analysis.

Figure 5d shows the estimated results of the longitudinal velocity. For the LS-GHCKF algorithm, it can be seen that the error between the estimated value and the reference value does not exceed 0.01 m/s. For the LS-SRGHCKF algorithm, the error between the estimated value and the reference value does not exceed 0.005 m/s, and the estimated results are relatively stable. Figure 5e and Figure 5f, respectively, show the estimated results of the lateral velocity and the yaw rate. Additionally, the LS-GHCKF algorithm and the LS-SRGHCKF algorithm can accurately estimate the lateral velocity and the yaw rate, but the LS-SRGHCKF algorithm has a better filtering effect than the LS-GHCKF algorithm.

Further comparing the estimated accuracy, the root-mean-square error (RMSE) is used to evaluate the accuracy of the simulation results. The formula for this RMSE is as follows:

(53) $$M=\sqrt{\frac{\sum_{i=1}^{n}{\left(\hat{\sigma }-\sigma_{0}\right)}^{2}}{n_{0}}}$$

where *M* is the root-mean-square error; *n*_0_ is the quantity of the data; $\hat{\sigma }$ is the evaluated value; and *σ_0_* is the reference value.

As shown in Table 2, the RMSE values of three groups of estimated values were compared and analyzed: The difference in longitudinal velocity $\hat{v}$*_x_* between the two algorithms is 0.0045, the difference in lateral velocity $\hat{v}$*_y_* is 0.0014, and the difference in yaw rate $\hat{\omega }$*_r_* is 0.0001. We know that the LS-SRGHCKF algorithm and the LS-GHCKF algorithm have similar estimation effects on yaw rate, according to Formulas (10) and (12), and the yaw rate is both the input of the measurement equation and the output of the state equation; therefore, the LS-GHCKF algorithm and the LS-AGHCKF algorithm can accurately track the measurement signal and estimate the yaw rate. However, there are gaps in the estimation of longitudinal velocity and lateral velocity. By contrast, the LS-SRGHCKF algorithm estimates the longitudinal velocity and the lateral velocity more accurately. In general, the RMSE values of the LS-SRGHCKF algorithm are smaller than the ones of the LS-GHCKF algorithm for all three groups of estimated values, indicating that the LS-SRGHCKF algorithm has higher estimated accuracy. Therefore, the simulation results of vehicle state estimation based on the LS-SRGHCKF algorithm are better in accuracy and filtering effect than the LS-GHCKF algorithm.

### 3.2. Slalom Simulation

**Figure 6:**
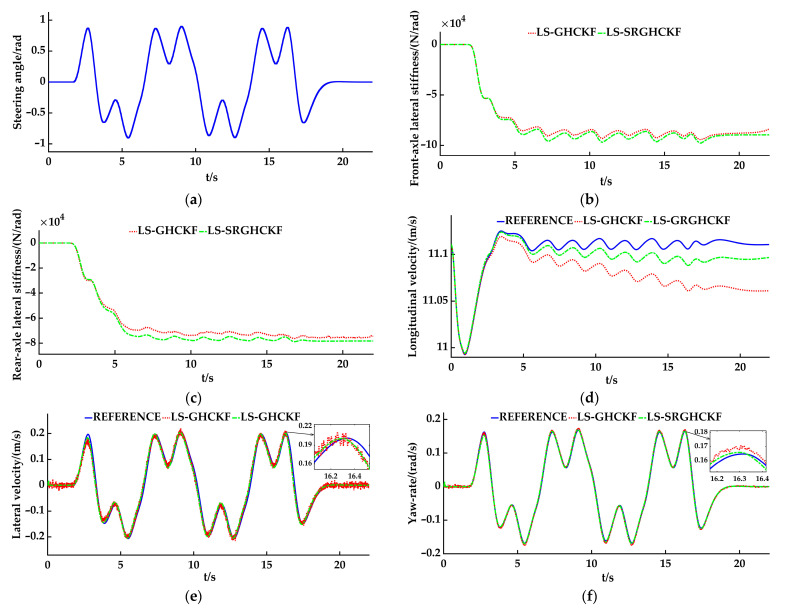
The simulation results of the slalom: (**a**) the steering angle; (**b**) the front-axle lateral stiffness; (**c**) the rear-axle lateral stiffness; (**d**) the longitudinal velocity; (**e**) the lateral velocity; (**f**) the yaw rate.

Regarding the low-speed slalom simulation working condition, the vehicle speed is set to 40 km/h, and the road adhesion coefficient *μ* is set to 0.85. The simulation step size is set to 0.005 s. Regarding the setting of the initial condition, the error covariance matrix **P** = 0.1 × eye(3), the process noise covariance matrix **Q** = 0.01 × eye (3), and the measurement noise covariance matrix **R** = 0.01 × eye (2). The simulation results are shown in Figure 6a–f, and the line type setting is the same as that in the double lane change simulation.

Analysis of the simulation results: Figure 6a shows the steering wheel angle-change curve during the vehicle’s running process along the slalom path. Figure 6b and Figure 6c, respectively, show the front- and rear-axle lateral stiffness curves based on the state variables of the vehicle state estimators, adopting the LS-GHCKF and LS-SRGHCKF algorithms. As shown in the figure, the estimated values of lateral stiffness in the two algorithmic environments are similar in size, and the trends of the data are basically the same. When the vehicle is running along the slalom path, compare Figure 6a with Figure 6b and Figure 6c. When the steering wheel angle curve shows peaks and valleys, the lateral stiffness curve also shows corresponding peaks and valleys, indicating that the lateral stiffness fluctuates with steering changes. After stable operation, overall, the average absolute value of the front-axle lateral stiffness of the two algorithms is about 89,000 N/rad, the average absolute value of the rear-axle lateral stiffness is about 74,800 N/rad, and the value of the rear-axle lateral stiffness is smaller than the value of the front-axle lateral stiffness. For the entire vehicle model used for simulation, the reason for the analysis is the same as that of the double lane change working condition.

Figure 6d shows the estimated results of the longitudinal velocity. The estimated accuracy of the LS-GHCKF algorithm and the LS-SRGHCKF algorithm is roughly the same as at the beginning. However, after the 3 s, the error begins to increase and stabilize until 16 s, where it reaches the maximum error value. The maximum difference between the estimated value of the LS-GHCKF algorithm and the reference value is 0.05 m/s, while the LS-SRGHCKF algorithm has a difference of 0.01 m/s. It can be seen that the LS-SRGHCKF algorithm has better stability and accuracy in estimating longitudinal velocity than the LS-GHCKF algorithm. Figure 6e and Figure 6f show, respectively, the estimated results of the lateral velocity and the yaw rate. Both algorithms can accurately estimate the lateral velocity and the yaw rate. From the locally enlarged image, it can be seen that the LS-GHCKF algorithm has a better filtering performance than the LS-GHCKF algorithm.

The RMSE is also used to evaluate the accuracy of the two algorithms, as shown in Table 3. The difference in longitudinal velocity $\hat{v}$*_x_* between the two algorithms is 0.0205, the difference in lateral velocity $\hat{v}$*_y_* is 0.0001, and the difference in yaw rate $\hat{\omega }$*_r_* is also 0.0001. We know that, except for the longitudinal velocity, the RMSE values of the LS-SRGHCKF algorithm and the GHCKF algorithm are very close and small, which illustrates that both algorithms have high accuracy in estimating the lateral velocity and the yaw rate. However, upon carefully comparing the two algorithms, the LS-SRGHCKF algorithm has obvious advantages in terms of accuracy and filtering.

## 4. Experimental Verification

**Figure 7:**
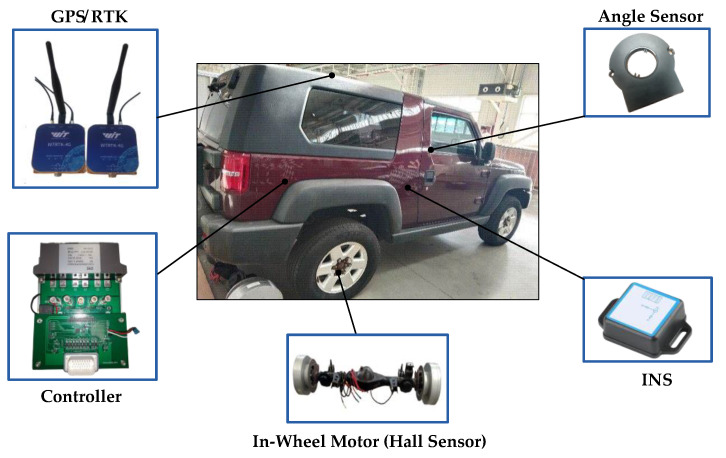
The vehicle and measurement sensors used in the experiment.

In order to further verify the effectiveness of the joint estimator and compare the progressiveness of the LS-GHCKF algorithm and the LS-SRGHCKF algorithm, a modified vehicle driven by a rear-drive, in-wheel motor was used to perform a road experiment. As shown in Figure 7, the angle sensor collects data on the steering wheel angle, the GPS/RTK module collects data on the vehicle’s longitudinal velocity and the heading angle, and the INS module collects data on the lateral acceleration, the lateral acceleration, the yaw rate, and the yaw angle. The lateral velocity cannot be directly measured, and integrating the measured lateral acceleration can easily cause cumulative errors. This article indirectly calculates the vehicle’s lateral velocity through the heading angle, the yaw angle, and the longitudinal velocity. The upper computer software collects and saves measurement data, imports them into the controller based on LS-SRGHCKF/GHCKF algorithms, and calculates the vehicle status values. As an intermediate variable, the lateral stiffness of the front and rear axle cannot be experimentally measured. Due to a detailed analysis of the lateral stiffness estimation process in the simulation, the final state estimation results of the vehicle were used in the experiment to indirectly verify the effectiveness of the joint estimator in estimating lateral stiffness.

### 4.1. The Double Lane Change Experiment

**Figure 8:**
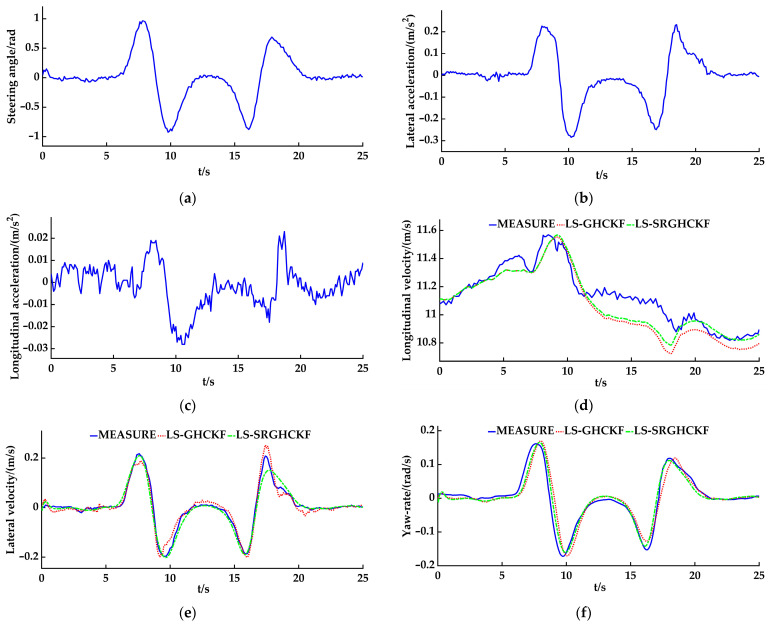
The experiment results of the double lane change: (**a**) the steering angle; (**b**) the lateral acceleration; (**c**) the longitudinal acceleration; (**d**) the longitudinal velocity; (**e**) the lateral velocity; (**f**) the yaw rate.

The double lane change experiment with a constant speed was conducted on an asphalt pavement (the coefficient of adhesion is 0.8–0.9). Considering the linear characteristics of the tires, the speed of the vehicle was set to 40 km/h, the sampling time interval of the angle sensor and INS module was set to 0.05 s, and the sampling time interval of the GPS/RTK module was set to 0.2 s. Figure 8a shows the data of the steering wheel angle, and Figure 8b and Figure 8c show, respectively, the lateral acceleration and the longitudinal velocity. To perform this procedure, researchers should import the measurement data into the LS-GHCKF and LS-SRGHCKF algorithm programs of the controller, calculate the values of the longitudinal velocity, the lateral velocity, and the yaw rate, and then compare the calculated values with the measurement data of the GPS/RTK/INS module. As shown in Figure 8d–f, the blue solid line represents the direct and indirect measurement data of the sensors, the red dashed line represents the estimated value of the LS-GHCKF algorithm, and the green dotted line represents the estimated value of the LS-SRGHCKF algorithm.

The experimental results show that the data of the steering wheel angle and lateral acceleration change according to the double lane change law and fluctuate with the entire vehicle’s movement. The longitudinal acceleration fluctuates at the 0-scale line. The changes in the steering wheel angle, lateral acceleration, and longitudinal acceleration are consistent with theoretical analysis and in line with actual working conditions. The LS-GHCKF and LS-SRGHCKF algorithms can track the longitudinal velocity of the vehicle, and both of them can perform effective filtering. The maximum relative error between the estimated and measured values of the LS-GHCKF algorithm is about 0.2 m/s, and the maximum relative error between the estimated and measured values of the LS-GHCKF algorithm is about 0.22 m/s. Therefore, the longitudinal estimated value of the LS-SRGHCKF algorithm is more accurate. From the figure, it can be observed that the lateral velocity estimation curve with the LS-SRGHCKF algorithm mostly fits the measurement curve and is very smooth. Therefore, compared to the LS-GHCKF algorithm, the LS-SRGHCKF algorithm is more accurate and has a better filtering performance. Due to the fact that the yaw rate serves as both an input of the measurement equation and output of the vehicle state estimator, the yaw rate values of the LS-GHCKF and LS-SRGHCKF algorithms basically overlap with the measurement data. However, the LS-SRGHCKF algorithm has higher accuracy and better filtering performance in some intervals than the LS-GHCKF algorithm, especially at the peak and valley positions of the curve.

RMSE is used to estimate the estimation accuracy. The RMSE for the experiment condition adopts Formula (53); at this point, *σ_0_* in the formula is the measurement value.

As shown in Table 4, the difference in the longitudinal velocity $\hat{v}$*_x_* between the two algorithms is 0.0187, the difference in the lateral velocity $\hat{v}$*_y_* is 0.001, and the difference in the yaw rate $\hat{\omega }$*_r_* is 0.0806. It can be seen that both the LS-GHCKF and LS-SRGHCKF algorithms have very small RMSE values, indicating that both algorithm estimates are valid and available. In a further comparison of RMSE between the two algorithms, the RMSE value of the LS-SRGHCKF algorithm is still smaller than that of the LS-GHCKF algorithm, suggesting that the estimated accuracy of the LS-SRGHCKF algorithm is higher. In summary, in the double lane change, low-speed experiment, the joint estimator based on the LS-SRGHCKF algorithm is significantly superior in terms of estimation accuracy and filtering effect compared to the one based on the LS-GHCKF algorithm.

### 4.2. The Slalom Experiment

**Figure 9:**
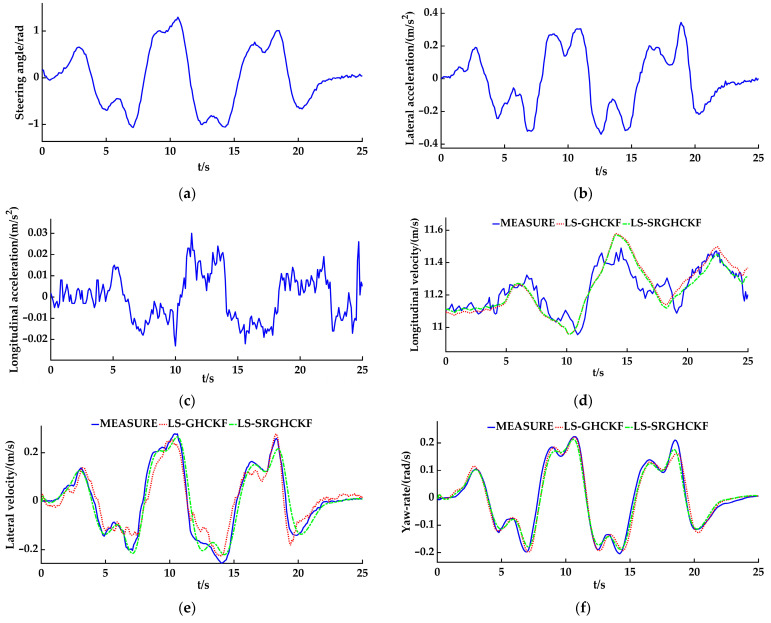
The experiment results of the slalom: (**a**) the steering angle; (**b**) the lateral acceleration; (**c**) the longitudinal acceleration; (**d**) the longitudinal velocity; (**e**) the lateral velocity; (**f**) the yaw rate.

A slalom experiment with a constant speed was conducted on an asphalt pavement (the coefficient of adhesion was 0.8–0.9). Considering the linear characteristics of the tires, the speed of the vehicle was set to 40 km/h, the sampling time interval of the angle sensor and INS module was set to 0.05 s, and the sampling time interval of the GPS/RTK module was set to 0.2 s. Figure 9 a shows the data of the steering wheel angle, and Figure 9b,c shows the lateral and longitudinal acceleration data. As with the double lane change experiment, researchers should import the measurement data into the LS-GHCKF and LS-SRGHCKF algorithm programs of the controller, calculate the longitudinal velocity, the lateral velocity, and the yaw rate, and then compare the calculated values with the measurement data of the GPS/RTK/INS module. As shown in Figure 9d–f, the line type setting is the same as that in the double lane change experiment curve.

The results show that, for estimating the longitudinal velocity, both the LS-SRGHCKF and LS-GHCKF algorithms can track the measurement curve well, but there is a significant difference between the two estimation algorithms after 14 s. However, visually, the LS-SRGHCKF estimation curve can better fit the measurement curve. For the lateral velocity, compared to the LS-GHCKF algorithm, the LS-SRGHCKF algorithm can track the trend of measurement curve changes; however, there are still errors. This phenomenon is mainly caused by measurement errors, model errors, and indirect calculation errors, and the LS-SRGHCKF algorithm has a significantly better filtering performance than the LS-GHCKF algorithm in estimating the lateral velocity. The estimated values of the LS-GHCKF and the LS-SRGHCKF algorithms can accurately estimate the value of the yaw rate, mainly because the yaw rate is both an input to the measurement equation and an output of the vehicle state estimator. However, when the vehicle turns, there is a relatively small error between the estimated and measured values, which is manifested as errors in peaks and valleys on the curve.

Further comparing the estimation accuracy, as in the double lane change experiment, the RMSE index is used for evaluation. As shown in Table 5, the difference in the longitudinal velocity $\hat{v}$*_x_* between the two algorithms is 0.0052, the difference in the lateral velocity $\hat{v}$*_y_* is 0.0095, and the difference in the yaw rate $\hat{\omega }$*_r_* is 0.0025. We know that the LS-SRGHCKF algorithm and the LS-GHCKF algorithm have similar estimation effects on the yaw rate. However, there is a gap in the estimation of the longitudinal velocity and the lateral velocity. In general, the RMSE values of both algorithms are very small, indicating that the estimated values of both algorithms are very close to the measured values and that the estimation results are effective. However, the RMSE value of the LS-SRGHCKF algorithm is smaller than that of the LS-GHCKF algorithm, indicating that the estimated accuracy of the LS-SRGHCKF algorithm is higher, especially for the estimation of the longitudinal velocity and the lateral velocity. Therefore, the LS-SRGHCKF has great advantages.

## 5. Conclusions

In this article, a vehicle state estimation method based on lateral stiffness is proposed. Advanced algorithms are used to design a joint vehicle state estimator. Simulation analysis and experimental methods are used to verify the joint estimator and its algorithm. The progressiveness of the technical route of the vehicle state is discussed, and the following conclusions are drawn:

A joint estimator that includes the lateral stiffness estimation and the vehicle state estimation can be implemented. Based on the establishment of a three-degree-of-freedom vehicle model and tire model, researchers designed the state equation and measurement equation of the joint state estimator and distinguished both the lateral acceleration and the yaw rate as two measurements; then, the principles of the lateral stiffness estimation and the vehicle state estimation were studied and analyzed. Furthermore, researchers designed a forgetting factor-based LS lateral stiffness estimator and a vehicle state estimator using the GHCKF/SRGHCKF algorithm.

Through simulation analysis, the lateral stiffness estimator based on the forgetting factor LS method can estimate the lateral stiffness of the vehicle. Both the LS-GHCKF algorithm and LS-SRGHCKF algorithm can estimate the longitudinal velocity, the lateral velocity, and the yaw rate of driving vehicles, with accurate and stable estimation results. The simulation results also indicate that the LS-SRGHCKF algorithm is significantly superior to the LS-GHCKF algorithm.

Through experimental verification, both the LS-GHCKF algorithm and the LS-SRGHCKF algorithm have obtained acceptable vehicle state estimation values. The experimental results are consistent with the simulation analysis results. Under different operating conditions, the LS-SRGHCKF algorithm has higher estimation accuracy than the LS-GHCKF algorithm, reflecting the effectiveness and robustness of the LS-SRGHCKF algorithm.

Further analysis of the simulation and experimental curves shows that the LS-SRGHCKF algorithm has a smaller estimated curve fluctuation than the GHCKF curve, indicating that the LS-SRGHCKF algorithm has a good filtering function. Through the in-depth comparison of RMSE values, it can be seen that the estimated RMSE of the LS-SRGHCKF algorithm is much smaller than that of LS-GHCKF, which indicates high accuracy in estimating vehicle states. In summary, the LS-SRGHCKF algorithm can stably and accurately estimate vehicle status, offering strong practicality and significant application value in the field of vehicle control.

## Figures and Tables

**Table 1:** Comparison and analysis of EKF, UKF, and CKF algorithms.

Kalman Algorithm	Principle	Application Scenario	Characteristic
EKF	Based on first-order Taylor series expansion; approximating nonlinear functions to linear functions	Generally applicable to weakly nonlinear systems.	The accuracy and stability of EKF for state estimation are also relatively average.
UKF	Using the traceless transformation method to approximate nonlinear functions	Generally applicable to strongly nonlinear systems.	When dealing with complex nonlinear systems, UKF usually has better performance than EKF.
CKF	Approximating nonlinear functions based on volume criterion	Can be applied to nonlinear systems with additive Gaussian white noise.	CKF has higher computational efficiency than UKF, and its approximation accuracy for nonlinear functions is lower than UKF.

**Table 2:** The RMSE of the double lane change simulation.

Estimated Value	LS-SRGHCKF_RMSE_	LS-GHCKF_RMSE_	DIFFERENCE
The longitudinal velocity $\hat{v}$*_x_*	0.0038	0.0083	0.0045
The lateral velocity $\hat{v}$*_y_*	0.0047	0.0061	0.0014
The yaw rate $\hat{\omega }$*_r_*	0.0019	0.0020	0.0001

**Table 3:** The RMSE of the slalom simulation.

Estimated Value	LS-SRGHCKF_RMSE_	LS-GHCKF_RMSE_	DIFFERENCE
The longitudinal velocity $\hat{v}$*_x_*	0.0122	0.0327	0.0205
The lateral velocity $\hat{v}$*_y_*	0.0012	0.0013	0.0001
The yaw rate $\hat{\omega }$*_r_*	0.0037	0.0038	0.0001

**Table 4:** The RMSE of the double lane change experiment.

Estimated Value	LS-SRGHCKF_RMSE_	LS-GHCKF_RMSE_	DIFFERENCE
$\mathrm{The}\;\mathrm{longitudinal}\;\mathrm{velocity}\;{\hat{v}}_{x}$	0.0926	0.1113	0.0187
$\mathrm{The}\;\mathrm{lateral}\;\mathrm{velocity}\;\hat{v}$ *_y_*	0.0171	0.0181	0.0010
$\mathrm{The}\;\mathrm{yaw}\;\mathrm{rate}\;\hat{\omega }$ *_r_*	0.0657	0.1463	0.0806

**Table 5:** The RMSE of the slalom experiment.

Estimated Value	LS-SRGHCKF_RMSE_	LS-GHCKF_RMSE_	DIFFERENCE
$\mathrm{The}\;\mathrm{longitudinal}\;\mathrm{velocity}\;\hat{v}$ *_x_*	0.0730	0.0782	0.0052
$\mathrm{The}\;\mathrm{lateral}\;\mathrm{velocity}\;\hat{v}$ *_y_*	0.0329	0.0424	0.0095
$\mathrm{The}\;\mathrm{yaw}\;\mathrm{rate}\;\hat{\omega }$ *_r_*	0.0197	0.0222	0.0025

## Data Availability

Not applicable.
